# Minimizing an Electron Flow to Molecular Oxygen in Photosynthetic Electron Transfer Chain: An Evolutionary View

**DOI:** 10.3389/fpls.2020.00211

**Published:** 2020-03-13

**Authors:** Marina A. Kozuleva, Boris N. Ivanov, Daria V. Vetoshkina, Maria M. Borisova-Mubarakshina

**Affiliations:** Institute of Basic Biological Problems (RAS), Pushchino, Russia

**Keywords:** photosystems, evolution, plastoquinone, phylloquinone, oxygen, reactive oxygen species

## Abstract

Recruitment of H_2_O as the final donor of electrons for light-governed reactions in photosynthesis has been an utmost breakthrough, bursting the evolution of life and leading to the accumulation of O_2_ molecules in the atmosphere. O_2_ molecule has a great potential to accept electrons from the components of the photosynthetic electron transfer chain (PETC) (so-called the Mehler reaction). Here we overview the Mehler reaction mechanisms, specifying the changes in the structure of the PETC of oxygenic phototrophs that probably had occurred as the result of evolutionary pressure to minimize the electron flow to O_2_. These changes are warranted by the fact that the efficient electron flow to O_2_ would decrease the quantum yield of photosynthesis. Moreover, the reduction of O_2_ leads to the formation of reactive oxygen species (ROS), namely, the superoxide anion radical and hydrogen peroxide, which cause oxidative stress to plant cells if they are accumulated at a significant amount. From another side, hydrogen peroxide acts as a signaling molecule. We particularly zoom in into the role of photosystem I (PSI) and the plastoquinone (PQ) pool in the Mehler reaction.

## Introduction

Mehler reaction is the major source of reactive oxygen species (ROS), such as O_2_^∙–^ and H_2_O_2_, in chloroplasts. During the Mehler reaction, O_2_ molecules serve as an alternative electron acceptor from the photosynthetic electron transfer chain (PETC), being a safety valve to release surplus electrons and thus alleviating the PETC over-reduction. This reaction also contributes to building up of ΔpH across the thylakoid membrane and produces a signaling messenger, H_2_O_2_, which is capable of initiating various signaling pathways (Ivanov et al., 2012). However, an efficient electron flow to O_2_ would decrease the photosynthetic quantum yield. Moreover, ROS, if not neutralized efficiently, lead to oxidative damage. Thus, the PETC evolution could have been guided toward minimizing and/or taking strong control over the Mehler reaction.

**FIGURE 1:**
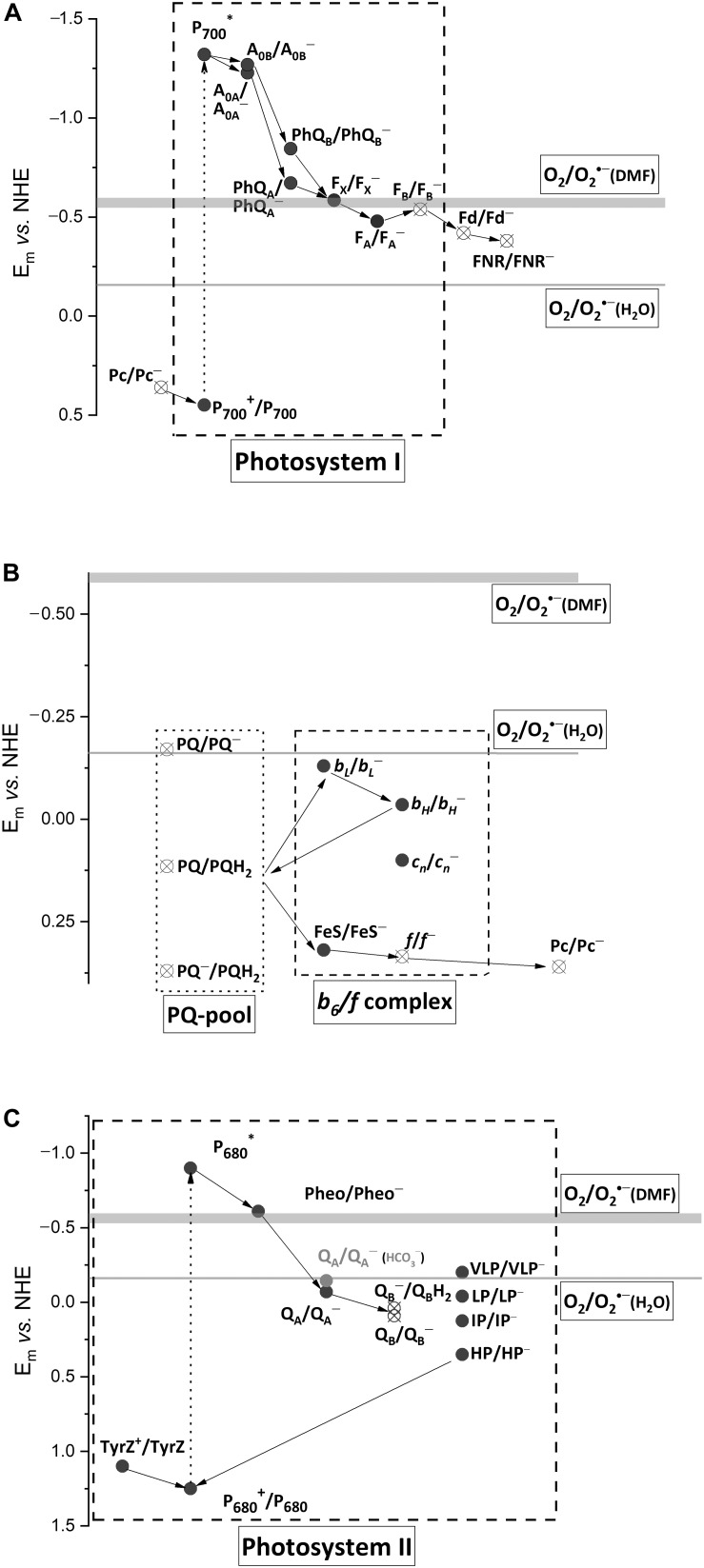
Schematic diagram of forward electron transfer in PSI **(A)**, PQ-pool and cytochrome *b_6_/f* complex **(B)**, and PSII **(C)**, with *E*_m_ values of the cofactors. The *E*_m_ values of (O_2_/O_2_^∙–^) in water, –160 mV, and dimethylformamide (DMF), from –550 to –600 mV, are shown by thin and thick horizontal lines, respectively. Pc, plastocyanin; P_700_, the dimer of Chl *a* molecules in PSI; A_0_, the primary electron acceptor in PSI; PhQ, phylloquinone, a secondary electron acceptor in PSI; F_X_, a 4Fe–4S cluster, a secondary electron acceptor in PSI; F_A_ and F_B_, 4Fe–4S clusters, the terminal electron acceptors in PSI; Fd, ferredoxin, the mobile electron acceptor; FNR, ferredoxin/NADP^+^ reductase; PQ, PQ^∙–^, and PQH_2_, plastoquinone, plastosemiquinone, and plastoquinol; FeS, a 2Fe-2S cluster of Rieske protein; *f*, cytochrome f; *b*_*L*_ and *b*_*H*_, the low- and high-potential forms of cytochrome *b*_6_; *c*_*n*_, the heme covalently bound to cytochrome *b*_6_; TyrZ, the redox active tyrosine residue; P_680_, the dimer of Chl *a* molecules in PSII; Pheo, pheophytin, a primary electron acceptor in PSII; Q_*A*_, the tightly bound plastoquinone in PSII, the secondary electron acceptor in PSII; Q_*B*_, the loosely bound plastoquinone in PSII, the terminal electron acceptor in PSII; VLP, LP, IP, and HP, the very low-, low-, intermediate-, and high-potential forms of cytochrome *b*_559_ in PSII. The *E*_*m*_-values for PSI cofactors are according to Ptushenko et al. (2008), those for *b_6_/f* complex are according to Alric et al. (2005), those for PSII are according to Brinkert et al. (2016) and Causmaecker et al. (2019), except cytochrome *b*_559_ (Khorobrykh, 2019). Closed circles, components situated in hydrophobic zones; open circles, water bulk phases exposed components.

Most of the PETC components were proposed as sites of O_2_^∙–^ photoproduction, the first step of the Mehler reaction. Among them, there are water-soluble and water-exposed components (Figure 1, open circles) and the components situated in hydrophobic zones (Figure 1, closed circles). The former produce O_2_^∙–^ in water bulk phases, e.g., stroma, while the latter produce O_2_^∙–^, which can be detected outside the membrane when diffused there or can be detected within the thylakoid membranes (Kozuleva et al., 2011). The value of *E*_*m*_ (O_2__/_O_2_^∙–^) in water is −160 mV, while in hydrophobic zones of proteins and membranes it is more negative, approximately −550 mV (Wardman, 1990). Only few components in PETC possess enough negative *E*_*m*_ for O_2_ reduction within a thylakoid membrane. Numerous experiments unambiguously demonstrated that photosystem I (PSI) is the major site of O_2_^∙–^ photoproduction (Kozuleva and Ivanov, 2016). O_2_^∙–^ generation by other components was shown under the disturbed PETC function. The second step of the Mehler reaction is H_2_O_2_ production via O_2_^∙–^ dismutation in stroma as catalyzed by superoxide dismutase. Apart from O_2_^∙–^ dismutation, another mechanism was shown to operate in the thylakoid membranes (Mubarakshina et al., 2006). It involves O_2_^∙–^ reduction by the plastoquinone (PQ) pool, namely, by plastoquinol (PQH_2_) (Borisova-Mubarakshina et al., 2019). Thus, the Mehler reaction proceeds at a variety of sites, still leading to O_2_^∙–^ and subsequent H_2_O_2_ production.

The evolution of various photosynthetic complexes has been a subject of several recent reviews (Jagannathan et al., 2012; Rutherford et al., 2012; Pierella Karlusich and Carrillo, 2017; Orf et al., 2018). Here, we briefly summarize the structural changes which could have happened in PETC to control and minimize an electron flow to O_2_. The general evolutionary trends could include: (i) kinetic control, making the forward reactions faster than the competing electron flow to O_2_, (ii) redox tuning of cofactors, disabling spontaneous exergonic reactions with O_2_, and (iii) shielding of cofactors with protein environment, restricting O_2_ accessibility (Rutherford et al., 2012).

## Photosystem I

All secondary electron acceptor cofactors of PSI were proposed as the sites of O_2_ photoreduction. The terminal FeS clusters F_*A*_/F_*B*_ are inevitably oxidized by O_2_ in the absence of ferredoxin (Fd). The role of intermediate cofactor FeS cluster F_*X*_ was claimed in Takahashi and Asada (1988) based on experiments showing that the primary H_2_O_2_ photoproduction site was a PsaA/PsaB heterodimer, which harbors F_*X*_. However, the PsaA/PsaB heterodimer also binds two phylloquinone (PhQ) molecules at the A_1_ sites and they could also contribute to H_2_O_2_ photoproduction. For the first time, the role of PhQs was proposed by Kruk with coauthors (Kruk et al., 2003) since adding PhQ to quinone-depleted thylakoid membranes re-established the O_2_ uptake at a single light flash. This result still does not rule out that FeS clusters reduce O_2_ by electrons from P_700_ *via* PhQ re-incorporated to the A_1_ sites. The PhQ involvement in O_2_ photoreduction in intact PSI under steady-state illumination was proposed based on comparing O_2_ photoreduction as a function of irradiance in the wild-type PSI with that in the mutant PQ-containing PSI (Kozuleva et al., 2014). The authors concluded that the PhQs at the A_1_ sites are the major contributor to O_2_^∙–^ generation.

From an evolutionary point of view, the terminal cofactor F_*B*_ can be one of the sites where the Mehler reaction should have been taken under control. This cofactor possesses negative *E*_*m*_, allowing for the efficient reduction of both Fd and O_2_. However, Fd is a mobile protein, diffusing to and out of PSI and leaving F_*B*_^–^ transiently open to O_2_. If F_*B*_^–^ is oxidized by O_2_ efficiently, it would be insufficient in a steady-state reduction of Fd. However, the electron lives mostly on F_*A*_, not F_*B*_, because of a positive shift of *E*_*m*_ (F_*A*_/F_*A*_^–^) relative to (F_*B*_/F_*B*_^–^) (Figure 1A; Fischer et al., 1997; Shinkarev et al., 2000). F_*A*_ is embedded deeper in the protein, which shields it from O_2_. This feature allows keeping of electrons for Fd and avoiding any wasteful electron leakage to O_2_.

The PsaC protein carrying F_*A*_ and F_*B*_ is homologous to mobile ferredoxins in anoxigenic phototrophs (Jagannathan and Golbeck, 2009). It is widely accepted that, during evolution, the ancestral mobile Fd was tightly bound to the ancestral homodimeric reaction center (RC). This binding resulted in an elongation of the ET chain in the RC that could have aimed at stabilizing the charge separation state and minimizing the charge recombination, which could lead to ^3^P_700_ and, hence, ^1^O_2_ formation (Orf et al., 2018). However, that binding probably provided an additional protein shielding for F_*X*_, which was the terminal cofactor in the ancestral RC, and for F_*A*_, limiting O_2_ diffusion and preventing unproductive electron leakage (Jagannathan et al., 2012). The protein shielding of these FeS clusters, being potentially capable of catalyzing H_2_O_2_ decomposition into a highly reactive HO^∙^ (Šnyrychová et al., 2006), could have additionally protected the PSI acceptor side from HO^∙^ formation.

Binding of the ancestral Fd to the ancestral homodimeric RC resulted in RC asymmetry through locating the F_*A*_ cluster closer to one of the quinones (PhQ_*B*_), bringing about a negative shift in *E*_*m*_ (PhQ_*B*_/PhQ_*B*_^∙–^) (Rutherford et al., 2012). The difference in *E*_*m*_ between PhQ_*A*_ and PhQ_*B*_ is up to 170 mV (Ptushenko et al., 2008). Rutherford with coauthors presented an elegant hypothesis explaining the benefit of this asymmetry as it eliminates ^3^P_700_ (and hence ^1^O_2_) formation under the conditions of the Fd pool over-reduction (Rutherford et al., 2012). In line with this hypothesis, PhQ^∙–^ oxidation by O_2_ sustains a forward ET and contributes to both alleviating PETC over-reduction and preventing charge recombination (Kozuleva and Ivanov, 2016). Both PhQs in PSI have one of the most negative *E*_*m*_ in the PETC (−671 and −840 mV for PhQ_*A*_ and PhQ_*B*_, respectively; Figure 1A), which allows phyllosemiquinones to reduce O_2_ even in the hydrophobic zones of the thylakoid membranes, where *E*_*m*_ (O_2_/O_2_^∙–^) is close to −550 mV (see above). Due to a longer lifetime, PhQ_*A*_^∙–^ gets higher chances to react with O_2_, although the more negative *E*_*m*_ of PhQ_*B*_/PhQ_*B*_^∙–^ provides a larger −Δ*G* in reaction with O_2_. However, the particular impact of each PhQ as well as clarifying the F_*X*_ role is still open questions.

## Ferredoxin and FNR

In bacterial type Fd, two 4Fe-4S clusters are partially exposed to solvent and accessible for O_2_ attacks (Jagannathan et al., 2012). After binding the ancestral Fd to RC, the organisms recruited another Fd, where a single 2Fe–2S cluster is shielded by a protein.

A long-lasting controversy on the role of Fd in the Mehler reaction was solved nearly a decade ago. In the absence of NADP^+^, which is the major electron sink for Fd, O_2_ inevitably oxidizes the reduced Fd (Fd^–^). In the presence of NADP^+^, simultaneously with its photoreduction, the electron flow to O_2_ was shown to be significant in high light; however, the contribution of Fd was almost negligible relative to that of the membrane-bound PETC components (Kozuleva and Ivanov, 2010). These results reveal a low reactivity of Fd^–^ toward O_2_, which enables Fd to fulfill the function of stromal hub-donating electrons to multiple enzymes and proteins, including ferredoxin-NADP^+^ reductase (FNR) (Hanke and Mulo, 2013).

The Fd affinity to its redox partners, i.e., PSI acceptor side, was also raised to ensure the competition with O_2_ for electrons. However, this is not entirely the case of FNR. Although a semiquinone form of FAD prosthetic group in FNR can react with O_2_ (Massey, 1994), so far there are no reliable experimental data demonstrating that FNR is involved in O_2_ photoreduction in the thylakoid membranes (Kozuleva and Ivanov, 2016). The FNR of oxygenic phototrophs possesses ∼10 times higher catalytic activity than the bacterial FNR (Pierella Karlusich and Carrillo, 2017), with affinity remaining roughly the same. The high catalytic activity is likely achieved through conformational changes caused by NADP^+^ binding to FNR, which greatly facilitate both the Fd^–^ oxidation (Batie and Kamin, 1984) and the liberation of the oxidized Fd from the complex (Mulo and Medina, 2017). This enhancement in the FNR catalytic activity most possibly decreased the chances for both the FAD semiquinone (Q^∙–^) oxidation by O_2_ and the formation of Fd:FNR^∙–^ complex in the absence of NADP^+^.

## Plastoquinone Pool

O_2_^∙–^ photoproduction by PQ^∙–^ in the PQ pool was demonstrated (Khorobrykh and Ivanov, 2002; Vetoshkina et al., 2017). However, the maximal O_2_^∙–^ production rates observed in the pool were 10 times lower than in the PSI.

While anoxygenic phototrophs use menaquinone (MQ) and ubiquinone (UQ), the oxygenic ones recruited PQ, a representative of a “more recent” group of quinones (Schoepp-Cothenet et al., 2009). MQ was probably the first quinone in ancient photosynthetic membranes. The rationale for replacing MQ with PQ is clear: the *E*_*m*_ values of (Q/Q^∙–^) and (Q/QH_2_) are ∼100 mV (Kishi et al., 2017) and ∼180 mV (Bergdoll et al., 2016), more negative for MQ than for PQ (Figure 1B). This means that PQ^∙–^ and the PQ pool itself in the reduced state are more stable in the presence of O_2_. Furthermore, pK_*a*_ (Q^∙–^/QH) for PQ is higher than for MQ, providing an easier protonation and, hence, a higher stability of plastosemiquinone (Hasegawa et al., 2017).

A possible rationale for choosing PQ instead of UQ in the PETC of oxygenic phototrophs is still vague. Firstly, the O_2_^∙–^ generation by free UQ^∙–^ in the mitochondria was discovered as early as in 80-s (Turrens et al., 1985). This reaction has long been considered as an important source of O_2_^∙–^ in animal cells. On the contrary, PQ^∙–^ in photosynthetic cells has little impact on O_2_^∙–^ production, as stated above. Secondly, PQH_2_ is more efficient as an antioxidant than UQH_2_ (Borisova-Mubarakshina et al., 2019), e.g., in lipid peroxidation prevention (Kruk et al., 1997). A consequence of higher antioxidant activity of PQH_2_ is its higher ability to reduce O_2_^∙–^ to H_2_O_2_. It was shown that the PQ pool in the thylakoid membranes (presumably PQH_2_) is indeed oxidized by O_2_^∙–^ (Borisova-Mubarakshina et al., 2018). Therefore, despite the low O_2_^∙–^-generating activity, the contribution of the PQ pool to the Mehler reaction can be essential due to the production of H_2_O_2_ from O_2_^∙–^. We hypothesize that ensuring the efficient transformation of O_2_^∙–^, which is generated by PSI, to H_2_O_2_ could be one of the evolutionary driving forces for the choice of PQ.

Replacing MQ with PQ as a mobile pool in the thylakoid membrane inevitably affected all of the complexes interacting with quinone. All cofactors in photosystem II (PSII) and cytochrome *b_6_/f* complexes have 110–150 mV more positive *E*_*m*_ values than in their MQ-based analogs (Schoepp-Cothenet et al., 2009; Bergdoll et al., 2016).

## Cytochrome *b_6_/f* Complex

The cytochrome *b_6_/f* complex is also considered to be an O_2_ photoreduction site (Taylor et al., 2018). The high *E*_*m*_ values of the *b_6_f* complex cofactors are a consequence of MQ replacement with PQ (Bergdoll et al., 2016). Among its ET cofactors, the *b*_*L*_ heme possesses one of the lowest *E*_*m*_, −130 mV (Alric et al., 2005). Thermodynamically, this heme can hardly reduce O_2_ since *E*_*m*_ (O_2_/O_2_^∙–^) in the membrane is close to −550 mV (Figure 1B, see above). The fast ET from *b*_*L*_ to *b*_*H*_ decreases the possibility of a *b*_*L*_ reaction with O_2_.

In several studies, PQ^∙–^ at the quinol-oxidizing (Q_*o*_) site of the complex is considered as the electron donor to O_2_. However, the concerted oxidation of PQH_2_ diminishes the PQ^∙–^ lifetime. If semiquinone is produced, it is either quickly oxidized by *b*_*L*_ heme or reduced by it, if the heme is pre-reduced. The dimer organization of the *b_6_/f* complex was proposed to lower the chances of O_2_^∙–^ generation at the Q_*o*_ site (Rutherford et al., 2012). In the *bc*_1_ complex, a spin–spin complex state between the semiquinone and the Rieske cluster was shown to suppress O_2_^∙–^ generation (Bujnowicz et al., 2019). This keeps up well with the experimental observations that PQ^∙–^ can reduce O_2_ once it leaves the Q_*o*_ pocket (Forquer et al., 2006), becoming a part of the pool (see above). It was demonstrated that O_2_^∙–^ production by the isolated *b_6_/f* complexes was 10 times higher than the one by the isolated *bc*_1_ complexes (Baniulis et al., 2013). This can be explained by an easier liberation of semiquinone from the Q_*o*_ site in the former case. It is important that, *in vivo*, such PQ^∙–^ would appear at the luminal side of the thylakoid membrane. The lumen pH determines the protonation of PQ^∙–^. Since PQH^∙^ has a lower chance to reduce O_2_, the lumen pH can regulate the O_2_^∙–^ production there.

The appearance of semiquinone at the quinone-reducing site (Q_*r*_) of the *bc*_1_ complex from purple bacteria was shown (Drachev et al., 1989). There are still no reliable data on semiquinone formation at the Q_*r*_ site of the *b_6_/f* complex. The double reduction of PQ occurs there when the second electron is transferred to the *b*_*H*_ heme (Ivanov, 1993). The residence of the first electron at the *b*_*H*_ heme can be a result of the *c*_*n*_ heme situated in close vicinity to the *b*_*H*_ in the *b_6_/f* complex.

## Photosystem II

Three major tasks could have been solved during the evolution of PSII: (i) the existence of highly oxidizing P680^+^, (ii) dealing with charge recombination leading to ^1^O_2_ production, and (iii) stabilization of Q_*B*_^–^ waiting for the second electron (Rutherford et al., 2012). O_2_^∙–^ production in PSII was shown many times (Pospíšil, 2012). Pheophytin (Pheo), Q_*A*_, Q_*B*_, and cytochrome *b*_559_ were suggested as the sites of O_2_ reduction to O_2_^∙–^, based presumably on the experiments with PSII complexes with disrupted function, e.g., after modifications of the water-oxidizing complex.

Although Pheo^–^ possesses *E*_*m*_, −610 mV (Rappaport et al., 2002), negative enough to reduce O_2_ even in hydrophobic media (Figure 1C), its lifetime is rather short (300 ps) such that it prevents the electron leakage to O_2_. This reaction with Q_*A*_^–^ (Ivanov and Khorobrykh, 2003; Pospíšil, 2012) is thermodynamically unfavorable due to a more positive *E*_*m*_ (Q_*A*_/Q_*A*_^–^), −70 mV (Brinkert et al., 2016), than *E*_*m*_ (O_2_/O_2_^∙–^). However, the binding of HCO_3_^–^ to non-heme Fe situated between the Q_*A*_ and the Q_*B*_ shifts *E*_*m*_ (Q_*A*_/Q_*A*_^–^) to −145 mV, making the electron leakage from Q_*A*_^–^ to O_2_ more probable. In contrast to Q_*A*_, Q_*B*_ undergoes two sequential reduction steps, meaning that Q_*B*_^–^ lives for a longer time waiting for the second electron. However, Q_*B*_^–^ is thermodynamically stable due to the positive *E*_*m*_ potentials (Causmaecker et al., 2019).

The role of a very low potential form of cytochrome *b*_559_ (*E*_*m*_ is −150 to −200 mV) in O_2_ reduction was also proposed (Khorobrykh, 2019). However, the fraction of this form is extremely small under normal conditions and increases only when the PSII functioning is severely perturbed. The *b*_559_ heme is embedded in the hydrophobic zone of the membrane; therefore, O_2_ photoreduction by *b*_559_ heme is thermodynamically unfavorable.

## Discussion

In this review, we briefly summarize some features of the modern PETC, which have evolved at the background of the Mehler reaction. The main site of O_2_^∙–^ generation is PSI. Several experiments revealed that PhQ could be the major contributor to this process (Kruk et al., 2003; Kozuleva et al., 2011, 2014). The reactivity of the FeS components with O_2_, especially F_*B*_ and Fd, was diminished by redox tuning and protein shielding. The recruitment of a high-potential PQ to the membrane quinone pool instead of a low-potential MQ was driven by the necessity to keep the pool in the reduced state under illumination in the presence of O_2_. Replacing MQ with PQ triggered a redox tuning of PSII and cytochrome *b_6_/f* complex cofactors, disabling, among other things, efficient O_2_^∙–^ generation in these complexes. The only MQ-based cofactor preserved in the modern PETC is PhQ, which is likely to be the main site of O_2_^∙–^ generation.

The stromal production of O_2_^∙–^ *via* Fd greatly increases if the NADP^+^ recovering in the Calvin–Benson–Bassham cycle is retarded, e.g., due to closed stomata. In the stroma, H_2_O_2_ is produced from O_2_^∙–^ under catalysis by superoxide dismutase. O_2_ reduction by PhQ^∙–^ can account for O_2_^∙–^ appearance within the thylakoid membrane (Kozuleva et al., 2011); however, a significant part of O_2_^∙–^ formed by PhQ^∙–^ still likely diffuses outside the membrane. Nevertheless, the increasing irradiance resulted in both a larger O_2_^∙–^ production just within the thylakoid membrane and a larger H_2_O_2_ production *via* O_2_^∙–^ reduction by PQH_2_, i.e., by the mechanism different from dismutation (Borisova-Mubarakshina et al., 2012).

Thus, in chloroplasts, H_2_O_2_ is produced *via* two distinct reactions in two distinct chloroplast compartments. We believe that this observation may be important for the understanding of H_2_O_2_-mediated signal transduction. The stromal H_2_O_2_, which might be considered as a messenger of NADP^+^/NADPH status, can oxidize thioredoxins (Hofmann, 2010; Netto and Antunes, 2016). Therefore, a temporary H_2_O_2_ accumulation in the stroma can affect the expression of chloroplast genes and/or the translation aimed at the fast adaptation of photosynthetic apparatus. H_2_O_2_ formed by the membrane PQ pool might be considered as a messenger of PETC status. It is important in terms of the PQ pool function as a central hub, of which the redox state represents a signal for both the chloroplast gene expression (Pfannschmidt et al., 2009) and the retrograde signaling pathways from the chloroplast to the nucleus (Pfannschmidt et al., 2003). For example, the PQ pool redox state initiates the changes in the PSII light-harvesting antenna size as a long-term acclimation to light conditions (Escoubas et al., 1995; Frigerio et al., 2007). We demonstrated that it is H_2_O_2_ rather than the PQ pool reduction state itself that is responsible for the antenna size reduction in high light (Borisova-Mubarakshina et al., 2015, 2019). Therefore, we suppose that a high potential of the PQ pool to form H_2_O_2_ in high light and under stress conditions could serve as evolutionarily set to signal about the PETC redox state to adjust to the ever-changing environmental conditions.

## Author Contributions

MK and MB-M designed the concept of the article. All authors contributed to the writing of the first draft and manuscript revision, and approved the submitted version. MK incorporated all inputs from the coauthors, reviewers, and editor.

## Conflict of Interest

The authors declare that the research was conducted in the absence of any commercial or financial relationships that could be construed as a potential conflict of interest.
